# Eye Movements Reveal Optimal Strategies for Analogical Reasoning

**DOI:** 10.3389/fpsyg.2017.00932

**Published:** 2017-06-02

**Authors:** Michael S. Vendetti, Ariel Starr, Elizabeth L. Johnson, Kiana Modavi, Silvia A. Bunge

**Affiliations:** ^1^Department of Psychology and Helen Wills Neuroscience Institute, University of California, BerkeleyBerkeley, CA, United States; ^2^Oracle CorporationRedwood City, CA, United States

**Keywords:** eye movements, problem solving strategies, analogical reasoning, individual differences

## Abstract

Analogical reasoning refers to the process of drawing inferences on the basis of the relational similarity between two domains. Although this complex cognitive ability has been the focus of inquiry for many years, most models rely on measures that cannot capture individuals' thought processes moment by moment. In the present study, we used participants' eye movements to investigate reasoning strategies in real time while solving visual propositional analogy problems (A:B::C:D). We included both a semantic and a perceptual lure on every trial to determine how these types of distracting information influence reasoning strategies. Participants spent more time fixating the analogy terms and the target relative to the other response choices, and made more saccades between the A and B items than between any other items. Participants' eyes were initially drawn to perceptual lures when looking at response choices, but they nonetheless performed the task accurately. We used participants' gaze sequences to classify each trial as representing one of three classic analogy problem solving strategies and related strategy usage to analogical reasoning performance. A project-first strategy, in which participants first extrapolate the relation between the AB pair and then generalize that relation for the C item, was both the most commonly used strategy as well as the optimal strategy for solving visual analogy problems. These findings provide new insight into the role of strategic processing in analogical problem solving.

## Introduction

Analogical reasoning—the process of generating inferences based on relational correspondences between two domains—is ubiquitous in most forms of thought (Hofstadter and Sander, 2013). Analogical reasoning is a powerful tool for acquiring new information, in that it enables learners to structure information from novel domains by forming parallels with known domains. As such, it is not surprising that analogical reasoning ability is a strong predictor of academic and professional achievement (Kuncel et al., 2004). Numerous models have been put forth to explain the processes involved in analogical reasoning (see Gentner and Forbus, 2011). Although most of these models share many core processes (e.g., mapping items based on shared roles), what differentiates them is the information that is considered most useful for comparison when generating inferences.

**Figure 1:**
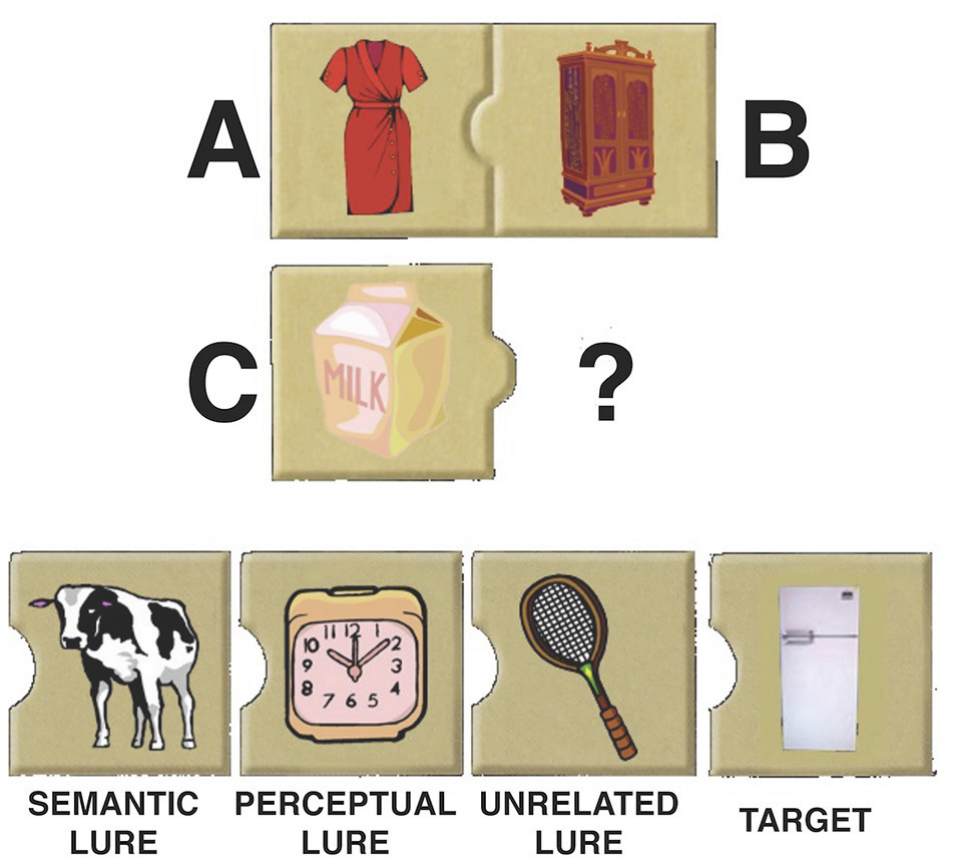
Example analogy trial. Participants were asked to choose which item best fills the position of the question mark. Each trial contained four response choices: the target, a perceptual lure, a semantic lure, and an unrelated lure. The position of each response choice was randomized across trials. Letters and the response choice labels are for illustrative purposes only.

Project-first models (e.g., Sternberg, 1977; Hummel and Holyoak, 1997; Doumas et al., 2008) stem from the psychometric tradition of using propositional analogies (i.e., A:B::C:?—see Figure 1) to study fluid intelligence. In these models, analogies are solved by first generating a rule that relates the A and B terms, then mapping the A and C terms, and finally applying a similar rule that generates D. According to this model, when presented with the analogy GLOVE: HAND:: SOCK: FOOT, one would first identify a rule relating glove and hand (e.g., a glove covers a hand for warmth). Then one would identify a rule that relates glove and sock (e.g., they are both articles of clothing). After mapping the rule between the two domains, one would arrive at a solution (e.g., a sock covers a foot for warmth). Thus, the project-first model focuses on generating a rule between the A and B terms to guide subsequent judgments.

Structure-mapping models (e.g., Gentner, 1983, 2010; Falkenhainer et al., 1989) assume that knowledge is structured hierarchically with relations connecting items, and that analogies are solved by mapping items from one structured representation to another. This strategy, which is also referred to as alignment-first (Gentner and Forbus, 2011), begins with aligning the A and C terms, rather than the A and B terms. Once this alignment is made, the next step is to align the B item with the target. Using the same analogy example, structure-mapping models propose that generating a rule to relate glove and sock would be the integral step; other inferences would then be guided by how well the two structures correspond. Therefore, while project-first models employ a top-down matching order that begins with establishing the relation between A and B and generalizing that relation to C, structure-mapping models employ a bottom-up matching order that begins with generating possible sets of correspondences between A and C and choosing one that fits with B (Gentner and Forbus, 2011).

A third model is based on the idea that the A and B terms in an analogy problem are related in multiple ways. Using the sample analogy above, a glove and hand may be related as “clothing used to warm body parts.” However, many other relations may exist between these two items depending on the context, such as “things that are smaller than a breadbox.” Therefore, many possible solutions for solving the analogy could influence one's ultimate decision. This model, referred to hereafter as the semantic-constraint model (e.g., Chalmers et al., 1992; Thibaut et al., 2011; Glady et al., 2012), assumes that mapping relations between one's search space and the C term in the analogy is the most useful step for solving an analogy. Note, however, that this strategy fails to prioritize either the A or B items, and may therefore be considered a less efficient or mature strategy. Indeed, young children frequently resort to this strategy (Thibaut et al., 2011). Because analogical reasoning is cognitively demanding, this strategy may be employed under conditions when holding multiple relations in working memory places too great a burden on cognitive load.

Most models of analogical reasoning are based on behavioral findings, including comparing word passages (e.g., Gick and Holyoak, 1983; Day and Gentner, 2007), complex visual scenes (e.g., Markman and Gentner, 1993; Richland et al., 2006), and propositional analogies (e.g., Sternberg, 1977; Cho et al., 2007; Krawczyk et al., 2008). One potential limitation of behavioral data, however, is its low temporal specificity. Recording a behavioral response can provide a measurement for the outcome of a trial, but it does not capture the strategy used to arrive at that response. An approach with finer temporal resolution that allows researchers to gain insight into real-time strategy use is eyetracking (e.g., Hodgson et al., 2000; Salvucci and Anderson, 2001; Hayes et al., 2011; von der Malsburg and Vasishth, 2011). This approach uses eye movements to infer what participants were thinking based on where they were looking while solving a task (Yarbus, 1967; Just and Carpenter, 1976; Rayner, 2009).

A few recent studies have used eyetracking to study analogical reasoning (Gordon and Moser, 2007; Thibaut et al., 2011, Glady et al., 2014, 2016; Thibaut and French, 2016). Thibaut et al. (2011), Glady et al. (2014), and Thibaut and French (2016) have found that when adults are solving A:B::C:D analogies, they most frequently start with the A and B items, then move on to fixating on C and the response choices. Children, on the other hand, tend to initially fixate on the C item, and make fewer fixations on the A and B items as compared to adults. These results suggest that adults may adopt a project-first strategy for solving the analogies, whereas children may be more inclined to adopt a semantic-constraint strategy. These studies included a distraction manipulation: half of the trials contained a semantic distractor item that was conceptually related to the C item, and the other half contained only unrelated response options. Although children's analogical reasoning performance suffered on trials that included a distractor, it is difficult to determine whether or how these factors influenced adults' analogical reasoning strategies due to the small number of trials administered and adults' ceiling-level performance.

In the current study, we used eyetracking to determine what information participants attended to while solving propositional analogy problems in the face of distracting information. Previous work in both developmental and patient populations, as well as work using computational models, suggests that inhibitory control is a key driver of successful analogical reasoning (e.g., Richland et al., 2006; Krawczyk et al., 2008; Morrison et al., 2010). To further probe the role of inhibitory control, we included distractor response options on every trial that were either semantically or perceptually related to the C item, in order to examine the degree to which these lures capture adults' attention. This design therefore enabled us to directly test the relative saliency of perceptually- and semantically-related distracting information by pitting them against one another and assessing the effect on adult participants' analogical reasoning strategies and performance.

**Figure 2:**
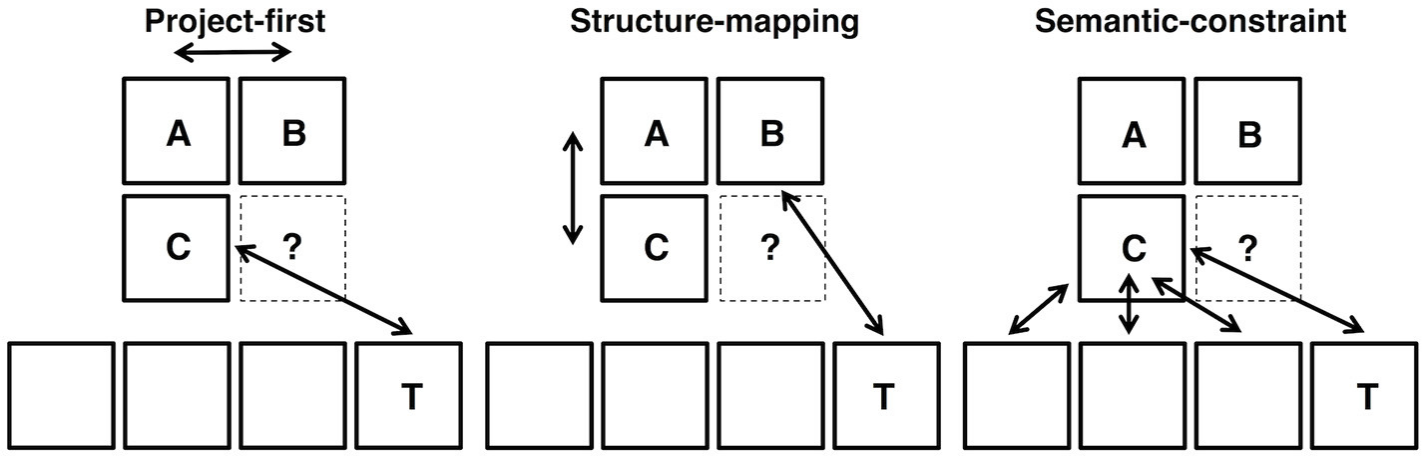
Patterns of eye movements as predicted by the project-first, structure-mapping, and semantic-constraint strategies.

A second goal of this study was to use patterns of eye movements on each trial to classify participants' trial-by-trial strategies as project-first, structure-mapping, or semantic-constraint. Each of these three strategies makes unique predictions regarding the patterns of eye movements used for solving analogy problems (Figure 2). According to the project-first strategy, solving analogy problems involves first focusing on the A and B terms of the analogy problem, and making fixation transitions between the A and B terms, before making fixations on and between C and the target. In contrast, the structure-mapping strategy predicts earlier fixations on the A and C terms, more transitions between the A and C terms, and then fixations on and between the B term and the target. Finally, the semantic association strategy predicts earlier fixations on the C term and response choices, as well as fixation transitions between these items. Therefore, participants' patterns of eye movements throughout a trial can provide insight into their analogy problem-solving strategies. We used the key distinctions between the models' predictions about eye gaze sequences, particularly with regards to which information is prioritized at the beginning of each trial, to classify strategy use on a trial-by-trial basis. Then, we investigated whether use of a particular strategy was predictive of overall analogy accuracy in order to determine if a particular strategy is optimal for analogical reasoning.

## Methods

### Participants

Twenty-eight healthy young adults (17 female, aged 18–25 years; mean 20.4 years; SD 2.01 years) participated in the study. All participants were university students, had normal vision, were fluent in English, had no reported history of neurological or psychiatric disorders, and gave their informed consent to participate in the experiment for partial fulfillment of a course requirement. The study was approved by the local ethics board. Two participants were excluded from the reported analyses: one whose proportion correct was more than two standard deviations below our sample's average performance and another whose eyetracking data was incomplete due to an error in data collection.

### Eyetracking apparatus

Stimuli were presented using the Tobii E-Prime Software Extensions (Psychology Software Tools, Pittsburgh, PA, 2012^1^), which synchronizes stimulus presentation timing with a second computer that records eye gaze position. Participants were seated comfortably in front of the Tobii T120 Eye Tracker (17-inch monitor, 1,280 × 1,024 pixel resolution). Distance was calibrated individually so that each participant focused on the middle of the screen, within a range of 50–80 cm. The Tobii T120 built-in camera captures data with a temporal resolution of 120 Hz and average spatial resolution of 0.3° of visual angle. The camera can automatically compensate for small head movements (within a 30 × 22 cm area at 70 cm distance); thus, participants' heads were not restrained. The camera independently recorded eye gaze position of the left and right eyes.

### Materials

Analogy trials were modeled off of the Matrices subtest of the Kaufman Brief Intelligence Test-Second Edition (Kaufman and Kaufman, 2004). Stimuli were designed using Adobe Photoshop, and made use of line drawings from “The Big Box of Art: 1 Million.” All stimuli were pictures of common objects. Analogy problems consisted of an incomplete propositional analogy (i.e., A:B::C:?) above a row of four items (Figure 1). Participants were asked to indicate with a button press which of the four response choices best completes the analogy. Participants were told that there may be several pictures that they think go with the C term, but that they should choose the picture that goes with the C term in the same way that the A term goes with the B term. Responses consisted of the correct response, a perceptual lure (an item that shared a similar shape and color to the C term), a semantic lure (an item whose meaning was associated with the C term, but did not match the relation shared between the A and B terms), and a lure that was designed to be unrelated both perceptually and semantically to the C term. The ordinal position of each of the four response options was randomized across trials.

### Testing procedure

Participants completed seven practice trials with feedback followed by 40 experimental trials without feedback. Experimental trials were split evenly into two blocks of trials. Each trial began on a black screen with a white central fixation cross. After 1,000 ms, an analogy problem was presented and remained on the screen until a response was made, or until the trial timed out after 10 s. Participants pressed a button on the keyboard to reflect which of the four response choices they thought best completed the analogy problem, and were instructed to respond as quickly and accurately as possible. The stimulus display then disappeared and participants were immediately presented with the next trial.

### Data analysis

Our analyses focused on fixations on and between seven critical areas of interest (AOIs): the A, B, and C items, as well as the Target, Semantic lure, Perceptual lure, and Unrelated lure items. We analyzed the duration of fixations occurring within each AOI, and the sequences of fixations between these AOIs. All analyses of eye movements were restricted to correctly performed trials. Fixations were classified using Tobii and trimmed to include only those between 150 and 1,000 ms to account for micro-movements and drift (Rayner et al., 2009).

#### Strategy classification algorithm

Each of the three strategies described above makes different predictions as to which fixations and fixation transitions are most informative for solving analogy problems. The project-first model states that initial fixations and transitions within the source domain (i.e., the A and B terms) are most useful for generating a relation to be applied between the C term and the target. Therefore, the project-first equation was defined as follows:

(1) $$\begin{array}{l} Project-First\;Strategy\text{ ​}=\text{ ​}Score_{initial\;fix.\;A}+\;Score_{initial\;fix.\;B}\\ \;+\;Score_{AB}+Score_{BA}+\;Score_{CT} \end{array}$$

where initial fixation corresponds to the first fixation occurrence in the sequence *for the specified AOI* (i.e., not necessarily the first fixation within the sequence), AB and BA correspond to fixation transitions between the A and B terms, and CT corresponds to fixation transitions from the C term to the target in the analogy problem. Under the project-first model, transitions are reciprocal between the A and B terms, but are specified as unidirectional from the C term to the target.

The structure-mapping model posits that initial fixations to, and transitions between, the A and C terms are most informative for generating a relation that should be applied between the B term and the target. As such, the structure-mapping equation was defined as follows:

(2) $$\begin{array}{l} Structure-Mapping\;Strategy=Score_{initial\;fix.\;A}\\ \;+\;Score_{initial\;fix.\;C}\text{​}+\text{​}Score_{AC}\\ \;+Score_{CA}+\;Score_{BT} \end{array}$$

Finally, the semantic-constraint model claims that relations between the C term and the response choices can be used to constrain the appropriate relation shared between the A and B terms in the analogy. Therefore, initial fixations to the C term and response choices, as well as bidirectional transitions, are most informative when solving the analogy problem. As such, the semantic-constraint equation was defined as follows:

(3) $$\begin{array}{l} Semantic\;-Constraint\;Strategy=Score_{initial\;fix.\;C}\\ \;+\;Score_{initial\;fix.\;T}+\;Score_{initial\;fix.\;S}\\ \;+Score_{initial\;fix.\;P}+Score_{initial\;fix.\;U}\\ \;+\;Score_{CS}+Score_{SC}+\;Score_{CP}\\ \;+Score_{PC}+\;Score_{CT}+\;Score_{TC} \end{array}$$

where S, P, and U correspond to the semantic, perceptual, and unrelated lure response choices, respectively.

For each correct trial, we used fixation AOI, fixation duration, and ordinal position within the fixation sequence to generate a score reflecting participants' strategy for that trial. The score is based on the assumption that fixations that occur earlier in a trial and those of a longer duration would occur within AOIs that are more useful for solving the analogy problem (cf. von der Malsburg and Vasishth, 2011). For each fixation in the fixation sequence for a trial, we calculated the score as the product of its duration and inverse of its location in the sequence. Therefore, longer fixations occurring earlier in the trial received higher scores, and shorter fixations occurring later in the trial received lower scores.

For each correct trial, a score was calculated for each strategy by summing up individual fixation scores for each of the fixation types present in the strategy equation. Once a score was calculated for each strategy based on the eye gaze sequence in a trial, we classified each trial as belonging to a particular strategy if its respective score was greater than the score generated by either of the other two strategies. Therefore, for a trial to be classified as project-first, the score for Equation 1 had to be greater than both the score from Equation 2 and the score from Equation 3. If the scores happened to be equal, no strategy classification would be given. Such an occurrence was rare, occurring in fewer than 4% of the trials included in the analysis (36 out of 934).

Our classification algorithm used a winner-take-all system in which the strategy with the highest score was assigned to that trial. To validate the algorithm, we trained a support vector machine classifier to sort trials into the three strategies. We used a six-fold cross validation method in which we split the dataset of correct trials into six even groups; for each classification attempt one of these folds was the test portion and the other five groups were the training set. The average accuracy of the multilabel classification (e.g., trials classified as project-first, structure-mapping, or semantic-constraint consistent with our algorithm) was 93 ± 6%, which suggests that our classification algorithm is acting on robust differences in eye gaze patterns that differentiate the strategies.

For each participant, we calculated the number of trials that were classified as each strategy type. To account for the slight variations in the number of trials included in the analysis across participants, we calculated the proportion of trials assigned to each strategy for each participant and used this proportion in follow-up analyses.

## Results

### Behavioral results

Participants performed the task well above chance (overall proportion correct: *M* = 0.91, *SD* = 0.08, response times on correct trials: mean = 3,078 ms, *SD* = 563 ms). This level of performance is similar to performance levels obtained in a previous study using similar analogy problems (Wright et al., 2008). Although participants performed well overall, when they did make errors they were most likely to choose the semantic lure (~64%), followed by perceptual (~26%), and unrelated lures (~10%). This pattern of errors was significantly different than what would be expected if error types were equally distributed [X^2^_(2)_ = 89.13, *p* < 0.001], indicating that participants were more likely to erroneously choose the semantic lure than either the perceptual or unrelated lure.

### Eyetracking results

The mean number of fixations per trial was 7.55 (*SD* = 3.41), and the mean fixation duration was 340.73 ms (*SD* = 168.88).

#### Proportion of time spent fixating AOIs.

**Figure 3:**
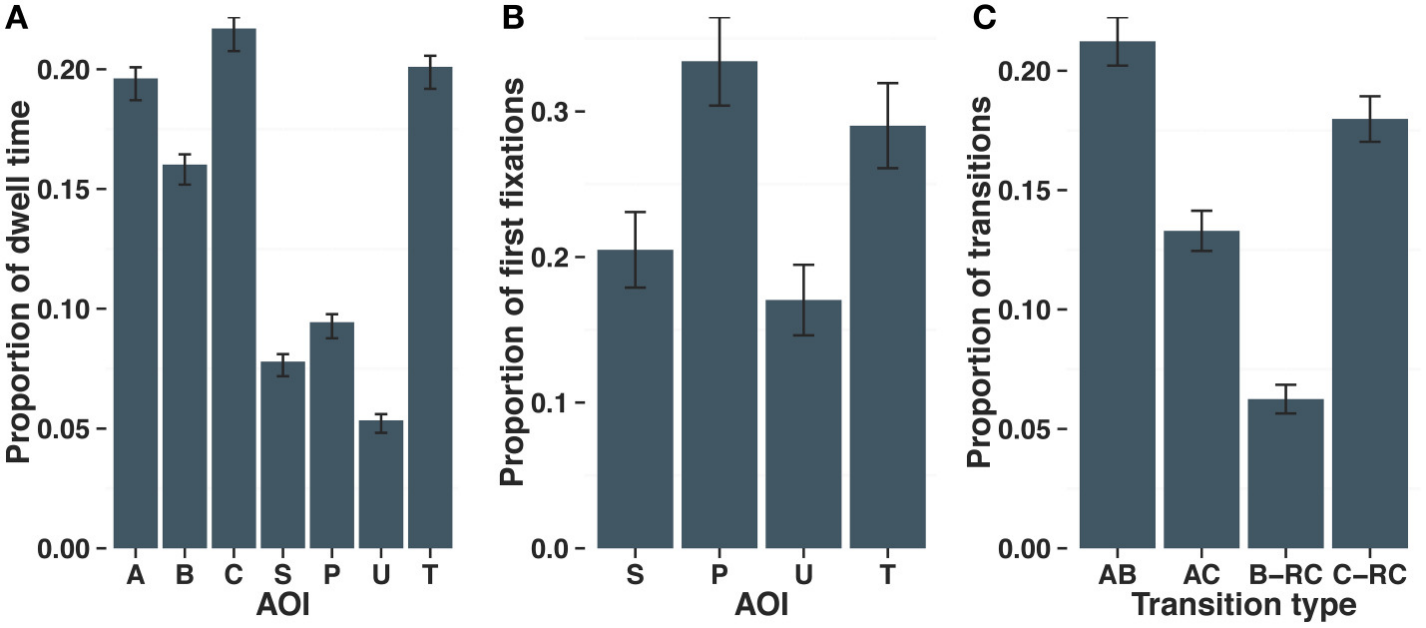
Proportion of dwell time in each AOI per trial **(A)**, proportion of first fixations on the response choices after having already fixated on the analogy terms **(B)**, and proportion of fixation transitions between AOIs of interest **(C)**. Error bars indicate 95% CI. S, Semantic lure; P, Perceptual lure; U, Unrelated lure; T, Target; RC, Response choices.

We measured the proportion of time that participants fixated each AOI (analogy terms: A, B, C; response choices: Target, Perceptual Lure, Semantic Lure, Unrelated Lure). There was a main effect of AOI on proportion of fixation duration [*F*_(6, 162)_ = 44.04, *p* < 0.001, $\eta_{p}^{2}$ = 0.44]. As shown in Figure 3A, participants spent significantly more time fixating on the A (*M* = 0.20, *SE* = 0.005), B (*M* = 0.16, *SE* = 0.09), and C (*M* = 0.22, *SE* = 0.004) terms as well as the Target (*M* = 0.20, *SE* = 0.005), relative to the Perceptual (*M* = 0.09, *SE* = 0.003), Semantic (*M* = 0.08, *SE* = 0.003), and Unrelated lures (*M* = 0.05, *SD* = 0.003).

#### First fixations among analogy terms

One important component underlying participants' strategy is the location of the first fixation when presented with the analogy problem. Participants fixated on the analogy terms prior to fixating on the response choices on the majority of trials (944 out of 985 trials, ~96%). For first fixations that did occur on an analogy term, there was a main effect of AOI on the location of the first fixation [*F*_(2, 54)_ = 24.28, *p* < 0.001, $\eta_{p}^{2}$ = 0.46]. Participants were significantly more likely to first fixate on the A and C items than the B item (*ts* > 6.5, *ps* < 0.001, *Cohen's Ds* > 1.23), with a trend toward more first fixations on the C item than the A item [*t*_(27)_ = 1.9, *p* = 0.07, *Cohen's D* = 0.35].

#### First fixations among response choices

We were also interested in testing whether participants were influenced by a particular lure when beginning their search among response choices. Because we were interested in how participants searched the response choices after attending to sample analogy items, we limited this analysis to participants' first fixations among the response choices that followed at least one fixation on either the A, B, or C item. The pattern of first fixations among response choices is shown in Figure 3B. There was a significant difference in the number of first fixations to each of the response choice AOIs [*F*_(3, 81)_ = 25.88, *p* < 0.001, $\eta_{p}^{2\;}$ = 0.40]. Participants made significantly more first fixations to the Perceptual Lure and Target compared to the Semantic and Unrelated Lures (ts > 5.0, *ps* < 0.001, *Cohen's Ds* > 0.95), with a trend toward more first fixations on the Perceptual Lure compared to the Target [*t*_(27)_ = 1.9, *p* = 0.07, *Cohen's D* = 0.36].

#### First-order fixation transitions

The next analysis concerned fixation transitions between AOIs. As described earlier, the three strategies predict different fixation transitions to be most useful while solving our analogy problems. Given the large number of possible saccades, we focused on saccades between A and B, A and C, B and the response choices, and C and the response choices. The strategy scores reflect the proportion of these transitions between AOIs rather than the absolute number of transitions. The distribution of the proportions of these transitions was significantly different than predicted by chance [*X*_(3)_ = 628.0, *p* < 0.001]. Participants made significantly more transitions between A and B (*M* = 0.21, *SE* = 0.005) compared to the number of transitions between A and C (*M* = 0.13, *SE* = 0.004), B and the response choices (*M* = 0.06, *SE* = 0.003), or C and the response choices (*M* = 0.18, *SE* = 0.005) (Figure 3C).

#### Using eyegaze sequences to identify optimal strategies

**Figure 4:**
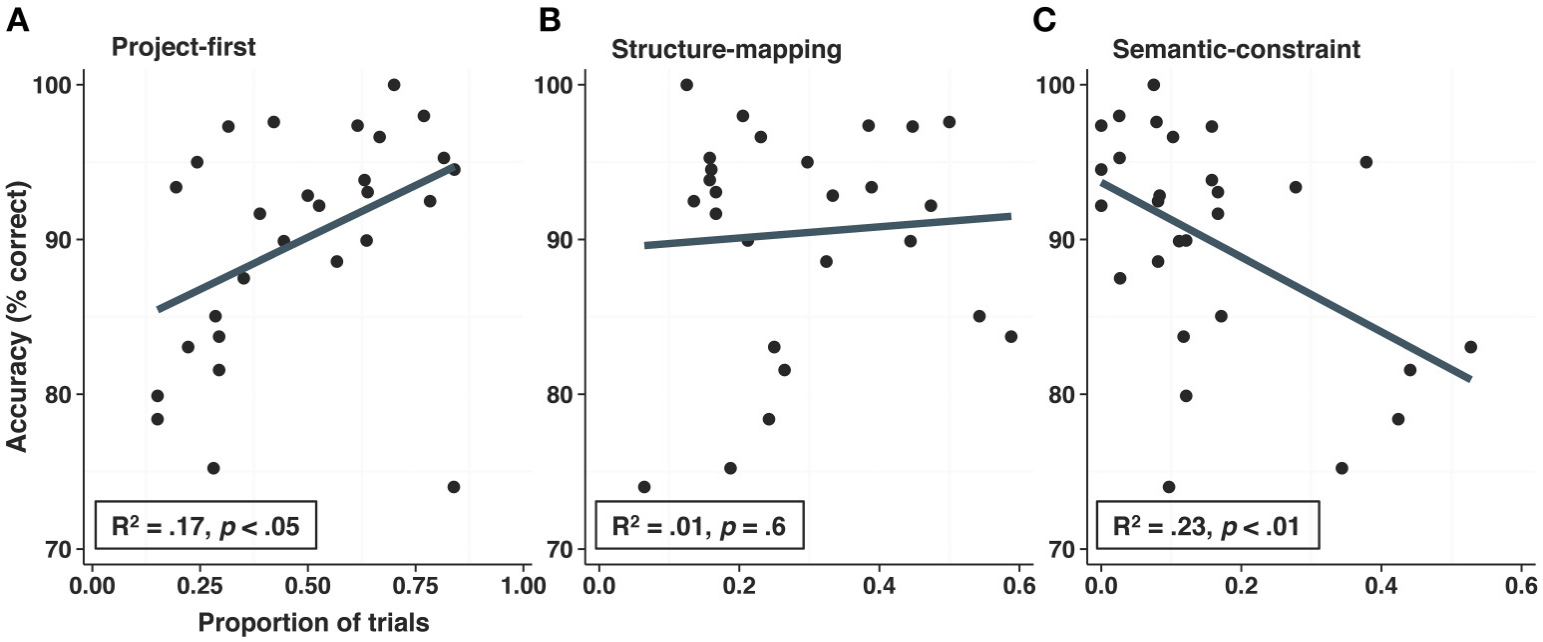
Correlations between strategy use and overall accuracy (mean percent correct). Use of the project-first strategy was positively correlated with accuracy **(A)**, whereas use of the structure-mapping strategy was unrelated to accuracy **(B)**, and use of semantic-constraint strategy was negatively correlated related with accuracy **(C)**.

Our strategy classification algorithm indicated that the majority of trials were classified as project-first (*n* = 446), followed by structure-mapping (*n* = 307), and semantic-constraint (*n* = 145). This distribution is significantly different than predicted by chance [*X*_(2)_ = 151.63, *p* < 0.001]. We next correlated participants' proportion of trials classified as each strategy with analogy accuracy (Figure 4). Despite the fact that accuracy was high for the group as a whole, the proportion of trials classified as project-first was positively correlated with accuracy [*r*_(26)_ = 0.42, *p* < 0.05]. By contrast, the proportion of structure-mapping trials was unrelated to accuracy [*r*_(26)_ = −0.09, *p* = 0.64], and the proportion of semantic-constraint trials was negatively correlated with accuracy [*r*_(26)_ = −0.48, *p* < 0.01]. These analyses indicate that a majority of the participants used the project-first strategy. Moreover, those who used it more often were more accurate, whereas those who used the semantic-constraint strategy more often were less accurate. Due to participants' overall high accuracy, incorrect trials were excluded from the strategy classification algorithm.

**Figure 5:**
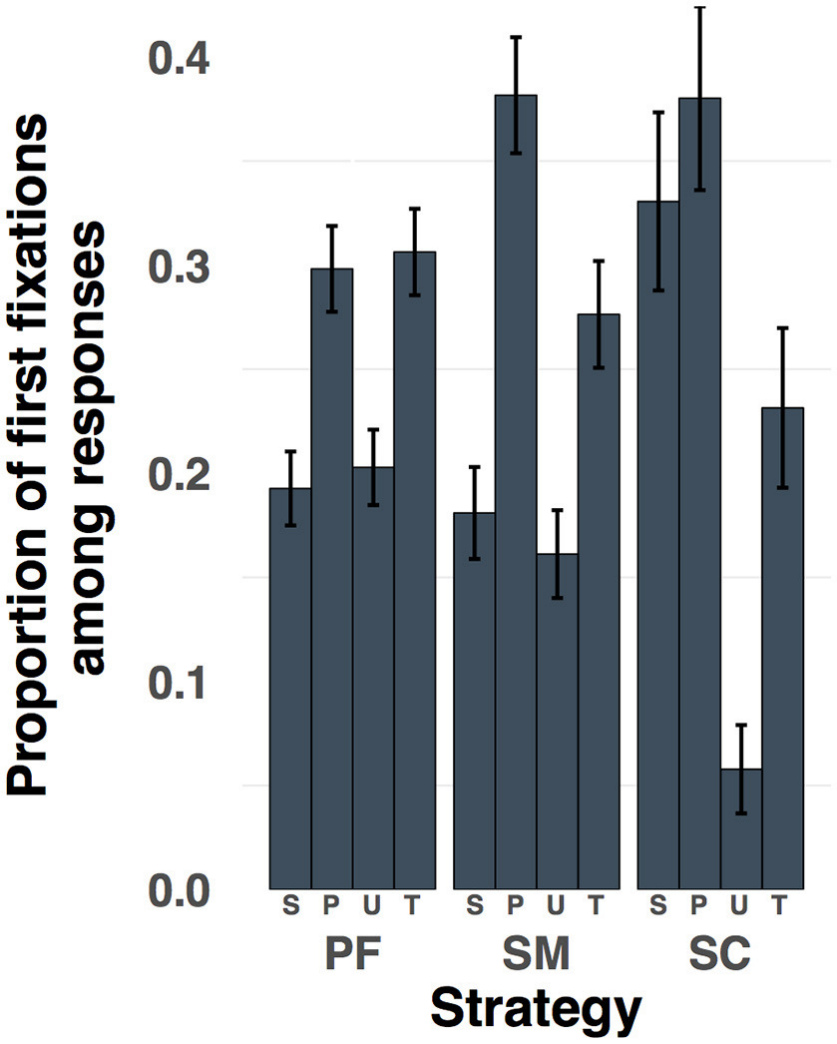
The proportion of first fixations on the response choices after having already fixated on the analogy terms, categorized by strategy. Error bars indicate 95% CI. S, Semantic lure; P, Perceptual lure; U, Unrelated lure; T, Target; RC, Response choices; PF, project-first; SM, structure-mapping; SC, semantic-constraint.

Strategy use also influenced participants' first response choice fixations, as indicated by a significant interaction between the first response choice fixation location and strategy [*F*_(6, 162)_ = 3.85, *p* < 0.005, $\eta_{p}^{2\;}$ = 0.04; Figure 5]. On trials in which participants used the project-first strategy, the first fixation among the response choices was equally likely to be on either the target or the perceptual lure [*t*_(27)_ = 0.295, *p* = 0.77, *Cohen's D* = 0.06]. However, on trials in which participants used the structure-mapping or semantic-constraint strategy, the first fixation was more likely to be on the perceptual lure than on the target response (*ts* > 2.0, *ps* < 0.05, *Cohen's Ds* > 0.39). In addition, only when participants used the semantic-constraint strategy were first fixations on the semantic lure as frequent as first fixations on the perceptual lure [semantic-constraint: *t*_(27)_ = −0.58, *p* = 0.57, *Cohen's D* = 0.11; project-first and structure-mapping: *ts* < −3.38, *ps* < 0.005, *Cohen's Ds* > 0.63]. Thus, on project-first trials participants were equally likely to first fixate on the perceptual lure or the target, but on structure-mapping trials there was a bias toward the perceptual lure whereas on semantic-constraint trials there was an increase in the proportion of first fixations on the semantic lure.

## Discussion

The aim of the current study was to use participants' eye movements to identify optimal strategies for solving visual analogy problems in the face of distracting information. Broadly, we sought to uncover how perceptual and relational similarity influence analogical judgments by determining which types of eye movements and fixations are used most often, and whether patterns of eye movements can predict one's task performance. To this end, we used participants' fixation sequences and corresponding fixation durations to calculate a score that was used to identify a strategy for each trial. We then calculated the proportion of trials assigned to each strategy and used this information to describe the distribution of strategies used across all trials and to predict participants' overall analogy accuracy.

Fixation patterns were classified on a trial-by-trial basis as representing one of three classic analogical problem solving strategies: (1) project-first (e.g., Sternberg, 1977), in which information between items in the source domain are used to generate a solution for the target domain; (2) structure-mapping (e.g., Gentner, 1983, 2010), in which comparing items between source and target domains is most fruitful for generating an analogy; and (3) semantic-constraint (e.g., Chalmers et al., 1992; Thibaut et al., 2010), in which participants rely on the semantic associations between the C term and response choices to guide their interpretation of the shared relation in the source domain. Each of these strategies predicts different steps that are required to solve analogy problems, and we leveraged participants' eye movements as a proxy of these steps.

Overall, we found that participants spent more time looking at the analogy terms and the target response, with much less time spent fixating on the incorrect response choices, including the semantic lure. Within each trial, participants' first fixation was most likely to be on the A or C item in the sample array. However, because the C item is centered within the stimulus array, it is not clear if the high proportion of first fixations on C reflects a true tendency for participants to begin with the C item, or if the proportion is inflated because participants are looking at the center of the screen when the trial begins. Nonetheless, we found that the most common fixation transition was between the A and B items. Further, our strategy classification analysis revealed that the project-first strategy, which prioritizes identifying the relation between the A and B items, was more common than either the structure-mapping or semantic-constraint strategies.

Notably, we also found that use of the project-first strategy correlated with overall analogical reasoning accuracy, such that participants who employed this strategy most often performed better than those who employed on it less frequently; by contrast, use of the structure-mapping strategy was not correlated with accuracy. These results are consistent with previous work suggesting that adults primarily focus on the AB relation (Thibaut et al., 2011; Thibaut and French, 2016) and provide empirical support for theory that this strategy is optimal for solving analogy problems (Hummel and Holyoak, 1997; Doumas et al., 2008).

In addition, we found that use of the semantic-constraint strategy, which initially focuses on finding a semantic relation for the C item, was negatively correlated with accuracy. This pattern of results resonates with previous work indicating that younger children, who are less proficient at solving analogy problems, demonstrate eye movements consistent with the semantic-constraint strategy (Thibaut et al., 2011; Glady et al., 2012). Thus, the semantic-constraint strategy represents a suboptimal and potentially immature approach to solving analogy problems.

Previous research on the process underlying visual analogy problem solving has frequently focused on the role of semantic interference by manipulating the number of semantic distractors included in the problems. These studies have found that, though performance in adults is unaffected by this manipulation, participants make more fixations to semantic distractors as the number of distractors increased (Gordon and Moser, 2007; Thibaut et al., 2010; Glady et al., 2014). The current study extended this work by including both perceptual and semantic distractors on each trial, thus allowing us to examine whether participants are influenced by perceptual similarity or are able to successfully ignore this task-irrelevant information, instead focusing on the relevant relational information.

We found that, although participants fixated most on the target item relative to the distractor items, the first fixation among the response choices was most frequently on the perceptual lure. This effect was driven by the trials in which participants used either the structure-mapping or semantic-constraint trials; on project-first trials, participants made an equal proportion of first response fixations to the target and the perceptual lure. Only when participants engaged in the semantic-constraint strategy did the semantic lure produce a similar attentional capture effect. In addition, though participants made more semantic than perceptual errors, they fixated equally often on the perceptual and semantic lures in correctly answered trials. These patterns demonstrate that perceptual similarity has a strong bottom-up influence on attention and suggest that perceptual similarity influences initial similarity judgments even if relational information ultimately guides response selection. Although participants were able to inhibit the impulse to select the perceptually similar item, they still found it to be a salient distractor. As such, this finding demonstrates that even high-performing adults initially make the types of similarity judgments that children demonstrate behaviorally (Gentner and Rattermann, 1991; Rattermann and Gentner, 1998). However, adults' high level of accuracy indicates that they are generally able to override any impulse to select a lure. This pattern of behavioral and eye gaze data provides support for the role of inhibitory control in mature analogical decision making, consistent with computational models such as LISA (Hummel and Holyoak, 2003).

Future research with this paradigm involving both children and adults could elucidate the extent to which developmental improvements in analogical reasoning hinge on increased inhibition of the tendency to respond on the basis of lower-level relations among stimuli, and/or increased use of the project-first strategy. In particular, manipulating the strength of the perceptual and semantic similarity between the distractors and the sample items could provide additional insights into the role of online inhibitory control in analogical reasoning. Another key direction for future research could be to explore the cognitive and environmental factors that contribute to differences in strategy use both across situations and across individuals. Ultimately, this work aims to delineate the necessary steps for effective reasoning in order to inform both theories of analogical reasoning as well as efforts to promote optimal reasoning strategies.

## Ethics statement

This study was carried out in accordance with the recommendations of the UC Berkeley Human Research Protection Program. All subjects gave written informed consent in accordance with the Declaration of Helsinki. The protocol was approved by the UC Berkeley Committee for Protection of Human Subjects.

## Author contributions

MV, EJ, and SB designed the study; EJ and KM collected the data; MV and AS analyzed the data; MV, AS, and SB wrote the manuscript.

### Conflict of interest statement

The authors declare that the research was conducted in the absence of any commercial or financial relationships that could be construed as a potential conflict of interest.

## Footnotes

^1^Psychology Software Tools and Inc, [E-Prime 2.0]. (2012). Available online at: http://www.pstnet.com
